# Pharmacokinetics of Intraperitoneal Vancomycin and Amikacin in Automated Peritoneal Dialysis Patients With Peritonitis

**DOI:** 10.3389/fphar.2021.658014

**Published:** 2021-05-28

**Authors:** Pâmela Falbo dos Reis, Pasqual Barretti, Laudilene Marinho, Andre Luís Balbi, Linda Awdishu, Daniela Ponce

**Affiliations:** ^1^ Internal Medicine Departament, University of São Paulo State–UNESP, São Paulo, Brazil; ^2^ UCSD Skaggs School of Pharmacy and Pharmaceutical Sciences, San Diego, CA, United States

**Keywords:** peritonitis, peritoneal dialiysis, amikacin, vancomicyn, pharmacokinetics

## Abstract

**Objective:** The study aimed to evaluate the vancomycin and amikacin concentrations in serum and dialysate for automatic peritoneal dialysis (APD) patients**.**

**Methods:** A total of 558 serum and dialysate samples of 12 episodes of gram-positive and 18 episodes of gram-negative peritonitis were included to investigate the relationship between vancomycin and amikacin concentrations in serum and dialysate on the first and third days of treatment. Samples were analysed 30, 120 min, and 48 h after intraperitoneal administration of vancomycin in peritonitis caused by gram-positive agents and 30, 120 min, and 24 h after intraperitoneal administration of amikacin in peritonitis caused by gram-negative agents. Vancomycin was administered every 72 h and amikacin once a day. The target therapeutic concentration of amikacin was 25–35 mg/l at the peak moment and 4–8 mg/l at the trough moment; and after 48 h for vancomycin, 15–20 mg/l at the trough moment.

**Results:** For peritonitis caused by gram-negative agents, at the peak moment, therapeutic levels of amikacin were reached in dialysate in 80.7% of patients with evolution to cure and in 50% of patients evaluated as non-cure (*p* = 0.05). At the trough moment, only 38% were in therapeutic concentrations in the dialysate in the cure group and 42.8% in the non-cure group (*p* = 1). Peak plasma concentrations were subtherapeutic in 98.4% of the samples in the cure group and in 100% of the non-cure group. At the trough moment, therapeutic concentrations were present in 74.4% of the cure group and 71.4% of the non-cure group (*p* = 1). Regarding vancomycin and among gram-positive agents, therapeutic levels were reached at the peak moment in 94% of the cure group and 6% of the non-cure group (*p* = 0.007). After 48 h, 56.8% of the cure group had a therapeutic serum concentration whereas for the non-cure group it was only 33.3% (*p* = 0.39).

**Conclusion:** Despite a small sample size, we demonstrated peak dialysate amikacin level and peak serum vancomycin level correlates well with Gram-negative and Gram positve peritonitis cure, respectively. It is suggested to study the antibiotics pharmacodynamics for a better understanding of therapeutic success in a larger sample.

## Introduction

Peritonitis is a frequent, major complication of peritoneal dialysis (PD) that results in considerable morbidity, technique failure and mortality (Barretti et al., 2007; Brown et al., 2011; Hsieh et al., 2015). The International Society for Peritoneal Dialysis (ISPD) guidelines for PD-related infections (Li et al., 2016) recommend that initial empirical antibiotic therapy of peritonitis should include vancomycin for gram-positive agents and aminoglycosides for gram-negative agents, via the intraperitoneal (IP) route for optimal efficacy (Li et al., 2016).

Vancomycin is recommended to be administered intermittently in an IP dose of 15–30 mg/kg body weight daily for 3–7 days, therefore monitoring the serum antibiotic levels and adjusting dosages may be important for maximizing therapeutic efficacy and minimizing toxicity (Li et al., 2016). However, the evidence underpinning these recommendations is sparse. In a retrospective investigation of 31 episodes of gram-positive PD-related peritonitis, Mulhern et al. (Mulhern et al., 1995) observed that an initial day-7 trough serum level of < 9 mg/l predicted a significantly increased risk of peritonitis relapse. In contrast, Blunden et al. (Blunden et al., 2007) reported that day-5 vancomycin levels did not predict cure or relapse of gram-positive or gram-negative peritonitis in 374 PD patients and that increasing the vancomycin concentrations did not appear to improve the cure rates.

One of the options for the treatment of gram-negative peritonitis is the use of aminoglycosides. These should be administered intermittently in a daily IP exchange, with a minimum stay of 6 h (Li et al., 2016).

Some studies show that the use of IP gentamicin can accelerate the loss of residual renal function, although this has not been demonstrated in one large multicentre study (Gendeh et al., 1993; Shemin et al., 1999; Badve et al., 2012). The ISPD guidelines recommend monitoring serum aminoglycoside levels when toxicity is suspected, although evidence of the benefit of monitoring is weak (Li et al., 2016).

Tang et al. (2014) evaluated 185 episodes of peritonitis caused by gram-negative agents treated with IP gentamicin. The serum concentration of the drug was assessed on the second and fifth days of treatment. No relationship was observed between cure and the serum gentamicin level.

Blunden et al. (2007) evaluated 534 episodes of peritonitis treated with vancomycin (25 mg/kg) and gentamicin (0.6 mg/kg). After the fifth day of treatment, the serum concentration of both drugs was analysed. The results showed that 88.2 and 85% of the patients on continuous ambulatory PD (CAPD) and automatic PD (APD), respectively, obtained an adequate serum concentration of vancomycin (> 12 mg/l). However, the serum vancomycin concentration was not associated with a cure outcome. Regarding the dosage of gentamicin, the serum concentration was similar among those who achieved cure compared with those who required cateter removal. The dialysate dosage and toxic effects of the drugs were not evaluated.

Studies that analyse the pharmacokinetics of vancomycin and gentamicin in the treatment of peritonitis are rare and the population studied is small (Bailie et al., 1992; Blunden et al., 2007; Johnson, 2011; Fish et al., 2012; Tang et al., 2014; Mancini and Piraino, 2019). There are no studies that have analysed the association between serum and dialysate concentrations of amikacin and the cure of peritonitis.

Thus, although vancomycin and aminoglycosides are widely used drugs in the treatment of PD-related peritonitis, studies involving the safety and efficacy of their use are still scarce and inconclusive. Therefore, this study is justified to evaluate the IP use of vancomycin and amikacin in the treatment of peritonitis in APD patients, analysing the prescribed dose, the serum and dialysate concentrations at peak and trough moments, and their possible implications for the outcome of peritonitis.

## Methods

### Patients

This is a prospective observational cohort study of anuric adult patients with APD-related peritonitis caused by gram negative agents (enterobacterium except *pseudomonas*) sensitive to amikacin or gram positive agents sensitive to vancomycin using IP amikacin or vancomycin during 2018 and 2019. The dose of amikacin was 2 mg/kg in the last infused bag, with daily infusion, and for vancomycin a 30 mg/kg loading dose and a maintenance dose of 25 mg/kg every 72 h, with a minimum stay of 6 h for. Both antibiotics were used until the fifth day of treatment and then doses were adjusted according to culture and antibiogram.

Patients younger than 18 years, pregnant, urine output higher than 100 ml/day, patients on CAPD, negative culture, fungal peritonitis and aetiological agents resistant to vancomycin and amikacin were excluded. We followed the ISPD 2016 recommendations for diagnostic and empirical therapy of APD-related peritonitis.

The study was approved by the local research ethics committee under CAAE 96384718.0.0000.5411. Patients or legal guardians gave their informed consent.

The data were obtained by the same researcher through consultation of medical records, from the initial prescription of antibiotic until the outcome of the peritonitis: cure or non-cure (i.e., refractoriness, recurrence, catheter withdrawal, method change or death associated with peritonitis). Demographic, clinical and laboratory data and the outcomes of peritonitis (cure or non-cure) were recorded.

### Study Protocol

Serum and dialysate samples were analysed 30, 120 min and 48 h after vancomycin IP administration and 30, 120 min and 24 h after amikacin IP administration. Vancomycin was administered every 72 h and amikacin once a day. Target therapeutic concentrations of amikacin were considered to be 25–35 mg/l, at the peak moment and 4–8 mg/l at the trough moment; and for vancomycin, 15–20 mg/l at the trough moment.

**FIGURE 1:**
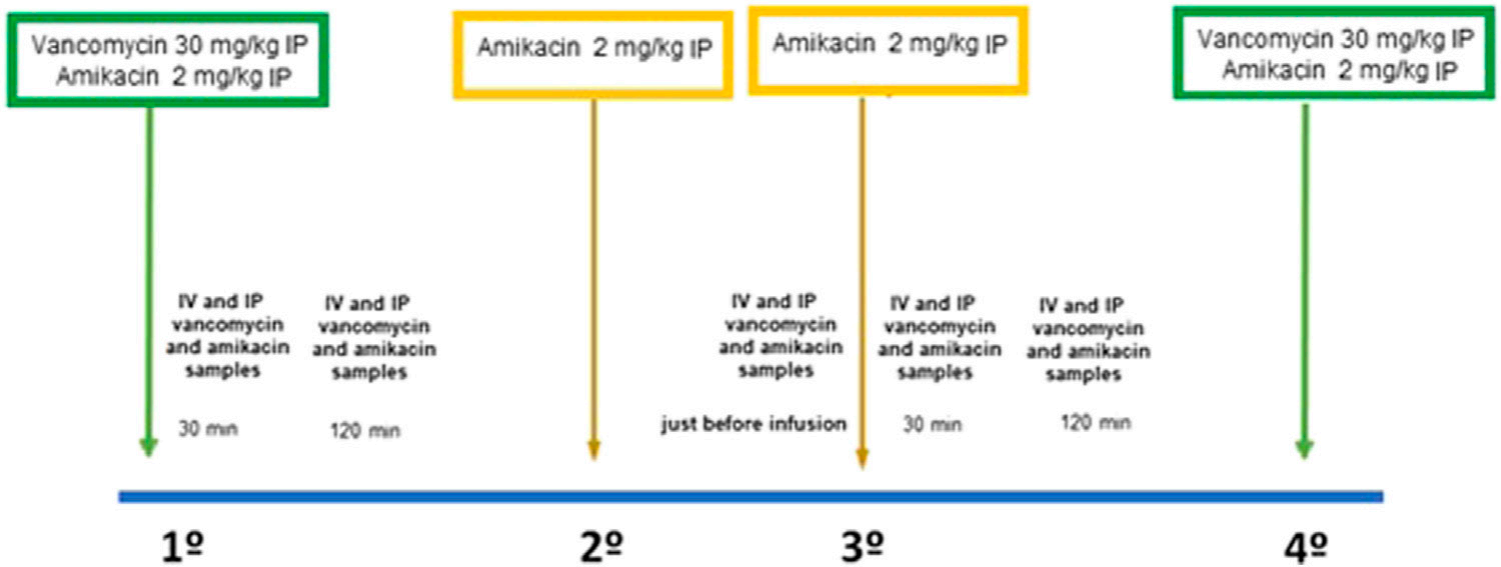
Timepoints when the serum and dialysate samples were collected and analysed.

Figure 1 shows the timepoints when the serum and dialysate samples were collected and analysed.

Serum vancomycin and dialysate analyses were performed with the VANC VITROS assay, using the VANC VITROS Chemistry Products reagent in conjunction with the VITROS Kit 11 calibrator in the VITROS 5.1 FS/4600 chemical system and the VITROS 5600 integrated system (Ortho Clinical Diagnostics, Rochester, NY, United States). VANC VITROS reagent is a double-chamber package containing liquid reagents that are used in a two-step reaction for the quantitative measurement of vancomycin. Reagent 1 is added to the sample to be tested, which contains vancomycin labeled with gluxose-6-phosphate dehydrogenase (G6PDH), followed by Reagent 2, which contains antibody against vancomycin, glucose-6-phosphate and adenine nicotinamide dinucleotide. The assay is based on the competition between vancomycin in the sample and vancomycin labeled with G6PDH at antibody binding sites.

After performing a calibration for each reagent lot, the concentration of vancomycin in each unknown sample can be determined using the stored calibration curve and the measured absorbance obtained in the sample assay.

Amikacin analyses were performed by particle-enhanced turbidimetric inhibition immunoassay, which is based on competition between the drug in the sample and the drug-coated microparticle for the antibody binding sites of anti-amikacin antibody reagente (Cerba Lab, Madri, Spain). The amikacin-coated microparticle reagent is rapidly agglutinated in the presence of the anti-amikacin antibody reagent and in the absence of any competing drugs in the sample. The rate of change in absorbance is measured photometrically, being directly proportional to the rate of agglutination of the particles. When a sample containing amikacin is added, the agglutination reaction is partially inhibited, reducing the rate of change in absorbance. A classic concentration-dependent agglutination inhibition curve can be obtained, with the maximum agglutination rate at the lowest amikacin concentration and the minimum agglutination rate at the highest amikacin concentration.

### Statistical Analysis

Analysis was performed using the SAS System for Windows statistical program (Version 9.2: SAS Institute, Cary, NC, United States) [EUA, 2012]. Initially, a descriptive analysis was performed for all patients and peritonitis episodes. Central tendency means, the dispersion of continuous variables and the frequency of categorical variables were calculated.

For comparison between groups, the chi-squared test was utilized for categorical variables and Student’s *t*-test or the Mann–Whitney test for continuous variables. Student’s *t*-test was used when the data were presented with a normal distribution; otherwise, the Mann–Whitney test was utilized. The dependent variable was defined as “cure of peritonitis”.

## Results

A total of 558 serum and dialysate samples of 30 episodes of peritonitis were evaluated: 12 episodes and 276 serum and dialysate samples of peritonitis caused by gram-positive agents (coagulase-negative staphylococci) and 18 episodes and 282 serum and dialysate samples of peritonitis caused by gram-negative agents (Enterobacterium, except *Pseudomonas*) during 2018 and 2019. Age was 60 ± 11.3 years, the main cause of kidney disease was diabetes (43.3%) and 63.3% were men (Table 1). The prescribed dose of amikacin was 2 ± 0.2 mg/kg and for vancomycin the loading dose was 26.9 ± 3.7 mg/kg and the maintenance dose was 15.32 ± 1.2 mg/kg every 72 h, with a minimum stay of 6 h.

**TABLE 1:** Clincial characteristics of APD patients-related peritonitis using IP vancomycin and amikacin during 2018 and 2019.

	*N* = 30
Age	60 ± 11.3
Male sex (%)	19 (63.3)
Main causse of CKD^a^ (%)	
Diabetes	13 (43.3)
Hypertension	4 (13.3)
Glomerulonephritis	9 (30.0)
Free time peritonitis (days)	340 (107.7–621)
Cell count 1 day^b^	1,090 (180–2,280)
Cell count 3 day^b^	80 (32–252)
Culture peritoneal effuentl (%)	
Gram-positive	12 (40)
Gram-negative	18 (60)

a CKD, chronic kidney disease.

b polymorphonuclear.

In peritonitis caused by gram-positive agents, serum vancomycin concentrations evaluated at 30, 120 min, and 48 h after administration were 20 ± 8.5, 23.3 ± 10.2, and 17.4 ± 7 mg/l, respectively; and vancomycin concentrations in the dialysate were 392.7 (5–897.2), 333.5 (6.6–661), and 7.2 (5–8.9) mg/l at 30, 120 min, and 48 h after IP administration. In peritonitis caused by gram-negative agents, serum concentrations of amikacin evaluated at 30, 120 min, and 24 h after administration were 6.4 (4–9.3), 10.2 (6.8–11.3), and 5.9 (3.6–9.5) mg/l, respectively, whereas amikacin concentrations were 3.2 (2.6–4.5), 51.1 (25.4–78.9), and 48.1 (36.6–90.2) mg/l at 30, 120 min, and 24 h after IP administration, as shown in Table 1.

There was cure in 83.3% of the episodes (there was non-cure in two episodes caused by gram positive and three by gram negative). When comparing characteristics between groups that evaluated cure and non-cure, there was no statistically significant difference in age, presence of diabetes, free time for peritonitis, cell count in the first day or aetiological agent. Cell count on the third day was associated with non-cure (*p* = 0.009), as shown in Table 1.

For peritonitis caused by gram-positive agents, the serum vancomycin concentration after 30 min of antibiotic infusion in the peritoneal cavity was a predictor of cure (*p* = 0.01), whereas for peritonitis caused by gram-negative agents there was an association between cure and amikacin concentration in the dialysate after 30 min. Serum and dialysate concentrations of amikacin and vancomycin at other timepoints were not associated with cure, as shown in Tables 2, 3.

**TABLE 2:** Analysis of serum concentrations and of vancomycin and amikacin dialysate at different moments after drugs intraperitoneal administration during peritonitis treatment.

	After 30 minutos *n* = 118	After 120 minutos *n* = 56	After 48 horas *n* = 102	p
Serum Vancomycin (mg/L)	20 ± 8.5	23.3 ± 10.2	17.4 ± 7	0.028
Vancomycin in dialysate (mg/L)	892.7 (5–1,297.2)	433.5 (6.6–661)	7.2 (5–8.9)	0.002
–	After 30 minutos *n* = 132	After 120 minutos *n* = 50	After 24 horas *n* = 100	p
Serum Amikacin (mg/L)	6.4 (4–9.3)	10,2 (6.8–11.3)	5.9 (3.6–9.5)	0.001
Amikacin in dialysate (mg/L)	51.1(25.4–78.9)	48.1 (36.6–90.2)	3.2 (2.6–4.5)	0.001

**TABLE 3:** Clinical and laboratorial characteristics of APD patients-related peritonitis and serum and dialysate concentrations after drugs intraperitoneal administration according to etiologic agent and cure and non-cure.

	Cure N^a^ = 25	Non-cure N^a^ = 5	p
Age	60.5 (55.5–64)	61 (55.75–70)	0.70
Diabetes (%)	10 (40)	3 (60)	0.36
Free time to peritonits (days)	345 (146–675)	107 (50.75–686)	0.53
Cell count (day 1)	1,200 (177,5–2,240)	980 (643.5–7,340)	0.46
Cell count (day 3)	77.5 (26.5–145)	1765 (435–2,960)	0.009
gram-positive agent	10 (40)	2 (40)	0.13
gram-negative agent	15 (60)	3 (60)	0.36
Gram-positive agent	N^a^ = 10	N^a^ = 2	
	^b^182 samples	^b^ 38 samples	
loading vancomycin dose (mg/kg)	27.1 ± 3.7	26.5 ± 4	0.761
maintenance vancomycin dose (mg/kg)	15 ± 2.1	15.15 ± 2.3	0.303
Serum concentrations of vancomycin (mg/L)			
30 min after IP administration	19.6 (13.9–25.3)	12.6 (10.2–16.5)	0.01
48 h after IP administration	17 (12.3–20.6)	13.4 (10.8–19.7)	0.52
Dialysate concentrations of vancomycin (mg/L)			
30 min after IP administration	1,121.6 ± 909.7	506.4 ± 318.5	0.1
			1
48 h after IP administration	6.9 (5–8.8)	7.7(5.6–8.6)	0.93
Gram-negative agent	N^a^ = 15	N^a^ = 3	
	^b^ 184 samples	^b^48 samples	
Amikacin dose	1.9 ± 0.2	1.9 ± 0.15	0.41
Serum concentrations of amikacin (mg/L)			
30 min after IP administration	6.4 (4.3–9.6)	6.5 (2.8–8)	0.31
24 h after IP administration	6 (3.7–9.7)	4.7 (3.8–6.6)	0.41
Dialysate concentrations of amikacin (mg/L):	N^b^ = 188	N^b^ = 44	
30 min after IP administration	50.3 (37.2–79.3)	23.5 (19–52.6)	0.02
24 h after IP administration	3.2 (2.6–4.8)	3.9 (3.6–5.2)	0.18

N^a^ = number of patients.

N^b^ = number of serum and dialysate samples.

**FIGURE 2:**
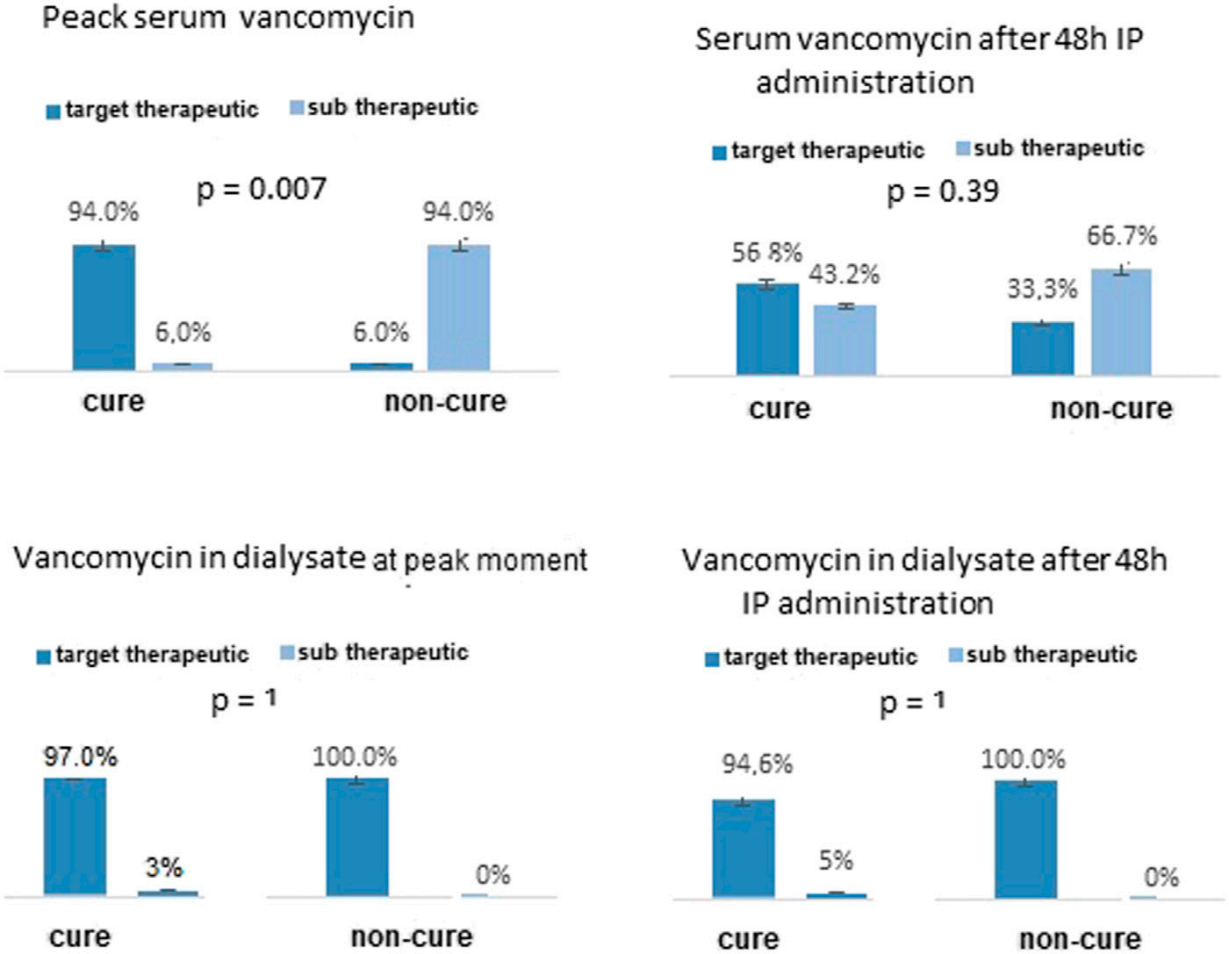
Analysis of serum and dialysate concentrations of vancomycin in APD patients-related peritonitis caused by gram positive agents at peak moment and after 48h IP administration of vancomycin according to cure and non-cure outcome.

**FIGURE 3:**
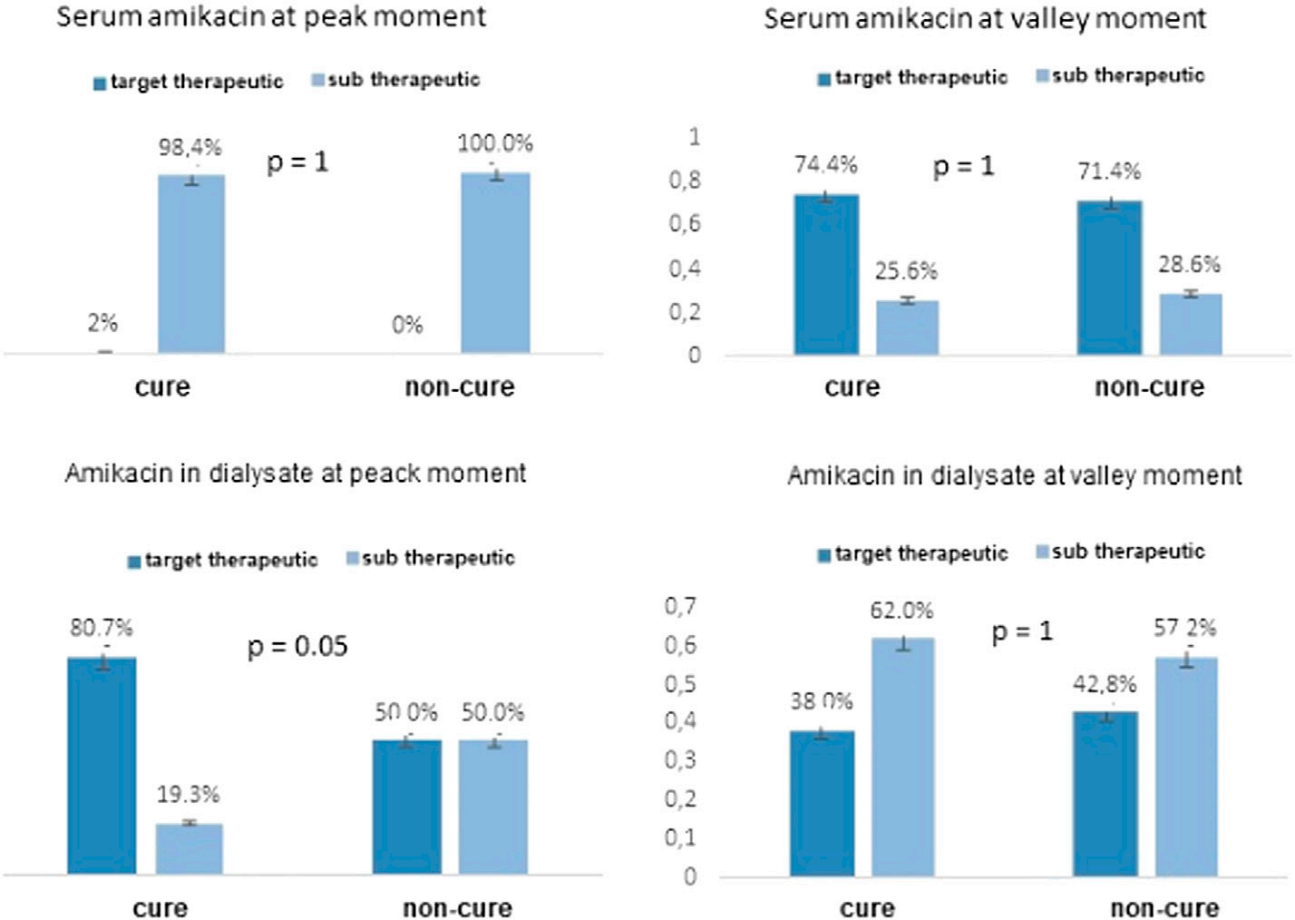
Analysis of serum and dialysate concentrations of amikacin in APD patients-related peritonitis caused by gram negative agents at peak moment and after 24h IP administration of amikacin according to cure and non-cure outcome.

Figure 2 and 3 shows the association between the peak concentrations and cure episodes. Among gram-negative episodes, at the peak moment, target therapeutic levels of amikacin were reached in 80.7% of patient dialysate samples with evolution to cure and in 50% of patients evaluated as non-cure (*p* = 0.05). Peak plasma concentrations were subtherapeutic in 98.4% of the samples in the cure group and 100% of the non-cure group. No association was observed at the trough moment between target therapeutic serum or dialysate concentrations and cure. Among gram-positive episodes, vancomycin therapeutic levels were reached at the peak moment in 94% of the cure group and 6% of the non-cure group (*p* = 0.007). After 48 h, 56.8% of the cure group and 33.3% of the non-cure group had a serum therapeutic concentration in the plasma (*p* = 0.39). No association was observed after 48 h of infusion between target therapeutic serum or dialysate concentrations and cure.

## Discussion

Pharmacokinetic and pharmacodynamics studies of drugs in the PD population are scarce and recommendations for treatment with IP antibiotics for patients with peritonitis are based on observational studies, mostly without monitoring serum and IP drug concentrations (Morse et al., 1987; Szeto et al., 2017; Lam et al., 2020).

Despite the importance of the topic, the level of clinical evidence to correctly guide antibiotic treatment in patients with PD-related peritonitis is weak. The ISPD guidelines recommend that antibiotics preferably are administered intermittently or continuously via the IP route (Li et al., 2016; Szeto et al., 2017; Lam et al., 2020). When administered intermittently, in order to cure the infection, back-diffusion of the drug in the serum to the dialysate must occur during exchanges without the antibiotic (Johnson, 2011).

Most previous studies evaluated the pharmacokinetics of drugs in the CAPD population and few have included APD patients (Mulhern et al., 1995; Tang et al., 2014). In a recent review (Mancini and Piraino, 2019), Mancini shows that the doses of antibiotics for APD patients are based on studies carried out on CAPD patients, which can lead to the use of antibiotic underdoses for APD patients.

Our study evaluated 30 episodes of peritonitis in anuric patients urdergoing APD and aimed to evaluate the prevalence of therapeutic vancomycin and amikacin in plasma concentrations and dialysate, in addition to analysing the impact of their inadequate concentrations on cure and non-cure outcomes.

Literature data suggest that, even with adequate treatment, 20% of episodes of peritonitis are refractory (Jain and Blake, 2006; Barretti et al., 2012; de Moraes et al., 2012; Salzer, 2018). In the Brazilian Peritoneal Dialysis Multicenter Study (BRAZPD), 14.1% of the cases were refractory (de Moraes et al., 2012). A study published by Tang et al., (2014) evaluated only peritonitis caused by gram-negative agents and refractoriness was 36%. In our study, peritonitis was refractory in 20% of episodes and in 60% the aetiological agents were gram-negative.

An evaluation of the cure outcome of peritonitis has been carried out in some studies. In the study by Krishnan et al., (2002), the time undergoing PD and the maintenance of cell count over 100 cells after the fifth day of treatment was a predictor of non-cure of peritonitis. In the BRAZPD, age, presence of collagenosis and peritonitis caused by *Pseudomonas* spp. were independent variables associated with the non-cure of peritonitis (de Moraes et al., 2012).

Our study looked for associations between plasma and IP drug concentrations at different moments and the clinical outcome of peritonitis, classified as cure or non-cure. With regard to gram-negative aetiological agents, target therapeutic concentrations of amikacin in the dialysate after 30 min of IP administration were identified as predictive variables for cure; for gram-positive aetiological agents, target serum therapeutic concentrations of vancomycin after 30 min of IP administration were associated with cure episodes.

Regarding the serum assessment of antibiotic concentrations, the ISPD guidelines recommend plasma monitoring of aminoglycoside and vancomycin levels in case of suspected toxicity and not to assess the effectiveness of the treatment (Li et al., 2016). However, the evidence for this recommendation is weak and nothing is stated about IP concentrations.

In our study, 30 min after IP administration of the antibiotics, vancomycin reaches the therapeutic plasma concentration in most patients: 94% in the cure group and 6% in the non-cure group (*p* = 0.007). Serum amikacin concentrations were subtherapeutic for most patients in both groups (98.4% in the cure group and 100% in the non-cure group, *p* = 1). The target serum amikacin concentrations were reached only at the trough moment (after 24 h of administration and immediately before the next dose) in 74.4% of the cure group and 71.4% of the non-cure group (*p* = 1) but there was no association with cure.

For aminoglycosides, similar to our study, the studies that evaluated serum concentrations in the trough moment found no relationship between serum concentrations and cure of peritonitis.

The high rates of therapeutic failure of peritonitis caused by gram-negative agents observed in the different studies, even in the presence of the agent’s sensitivity to the aminoglycoside, can be explained by the therapeutic target in the dialysate not being reached after 30 min of IP administration, whereas the non-success of treatment of peritonitis caused by gram-positive agents sensitive to vancomycin can be explained by the non-serum target therapeutic concentration.

Mulhern et al. (1995) in a previous study suggests that a plasma concentration greater than 12 mg/l in the trough moment is a predictor of higher cure rates for peritonitis.

Our study has some limitations. Few episodes of peritonitis were included and few measurements were performed to determine the pharmacokinetics of the drugs. For a better evaluation of the pharmacokinetics of vancomycin, the ideal analysis should include samples at 30 min, 1, 2, 6, 24, 48, and 72 h after administration, and similarly for amikacin up to 24 h. However, this would mean a change in the routine of the dialysis unit and the patients, in addition to being very costly, all of which makes it impossible to perform. There was no available data on minimum inhibitory concentration.

Despite these limitations, our study was the first to assess the plasma and IP concentrations of amikacin in anuric APD patients and to show the importance of reaching the therapeutic concentration in the dialysate at the peak moment, which may explain the failure of treatment due to sensitivity of the agent to amikacin. Further studies are needed to explore the reasons for not reaching IP therapeutic concentrations at the peak moment, such as type of peritoneal transport, presence of residual renal function.

In conclusion, the assessment of amikacin and vancomycin concentrations in dialysate and plasma illustrates that, at the peak moment, therapeutic concentrations of amikacin are required in the dialysate for therapeutic success, whereas plasma vancomycin concentrations appear to be more important for the cure outcome. Therefore, future studies that evaluate the pharmacokinetics of these drugs are essential for better understanding and therapeutic success, as peritonitis is associated with high rates of morbidity and mortality in PD patients, being the main cause of failure of technique and transition to haemodialysis.

## Data Availability

The raw data supporting the conclusions of this article will be made available by the authors, without undue reservation.
